# Activity patterns in mammals: Circadian dominance challenged

**DOI:** 10.1371/journal.pbio.3000360

**Published:** 2019-07-15

**Authors:** David G. Hazlerigg, Nicholas J. C. Tyler

**Affiliations:** 1 Department of Arctic and Marine Biology, UiT The Arctic University of Norway, Tromsø, Norway; 2 Centre for Saami Studies, UiT The Arctic University of Norway, Tromsø, Norway; Charité - Universitätsmedizin Berlin, GERMANY

## Abstract

The evidence that diel patterns of physiology and behaviour in mammals are governed by circadian ‘clocks’ is based almost entirely on studies of nocturnal rodents. The emergent circadian paradigm, however, neglects the roles of energy metabolism and alimentary function (feeding and digestion) as determinants of activity pattern. The temporal control of activity varies widely across taxa, and ungulates, microtine rodents, and insectivores provide examples in which circadian timekeeping is vestigial. The nocturnal rodent/human paradigm of circadian organisation is unhelpful when considering the broader manifestation of activity patterns in mammals.

## Abstract 2

The evidence that daily patterns of physiology and behaviour in mammals are governed by circadian ‘clocks’ is based almost entirely on studies of nocturnal rodents. This Essay proposes that the nocturnal rodent/human paradigm of circadian rhythms is unhelpful when considering the broader manifestation of temporal organisation of activity in mammals.

It is widely held that daily patterns of physiology and behaviour in mammals are governed by the cell-autonomous rhythms of gene transcription that constitute circadian ‘clocks’ [1]. Circadian clocks have been identified and characterised in species ranging from cyanobacteria to humans, and circadian organisation is generally considered a ubiquitous controlling feature [2–5].

The empirical basis for this view, however, is surprisingly weak. Knowledge of circadian mechanisms stems from studies in model organisms in which the phenotype is prominent and, in mammals, is based almost entirely on studies in rats, mice, and hamsters. This is no coincidence: these small nocturnal rodents are cheap to maintain, perform well in the laboratory, and above all, display strong circadian organisation. Had this not been the case, they would not have been studied: they were selected as models of circadian function, not of their taxa.

The ascendancy of the circadian model has led to uncritical use of the term ‘circadian’. Identification of circadian organisation (sensu stricto) requires evidence of persistence—i.e., the demonstration that rhythms are expressed in the absence of external synchronising input (the so-called zeitgeber). Such evidence is normally sought by observing organisms such as humans and mice under constant conditions―most often continuous darkness or dim red light. The term ‘circadian’ is nevertheless frequently ascribed in scientific literature to rhythms recorded under daily cycles of light intensity. Such usage without evidence of endogenous drive renders the term ‘circadian’ synonymous with ‘24 h’ or ‘diel’ and therefore redundant.

Fig 1 shows examples of two species from distinct mammalian taxa (common vole [*Microtus arvalis*] and reindeer/caribou [*Rangifer tarandus* L.], hereafter *Rangifer*) in which circadian organisation is not evident. Occurrence of noncircadian temporal organisation like this should make us wonder what circumstances have promoted circadian dominance in some species—notably, those upon which the canon is founded.

**Fig 1:**
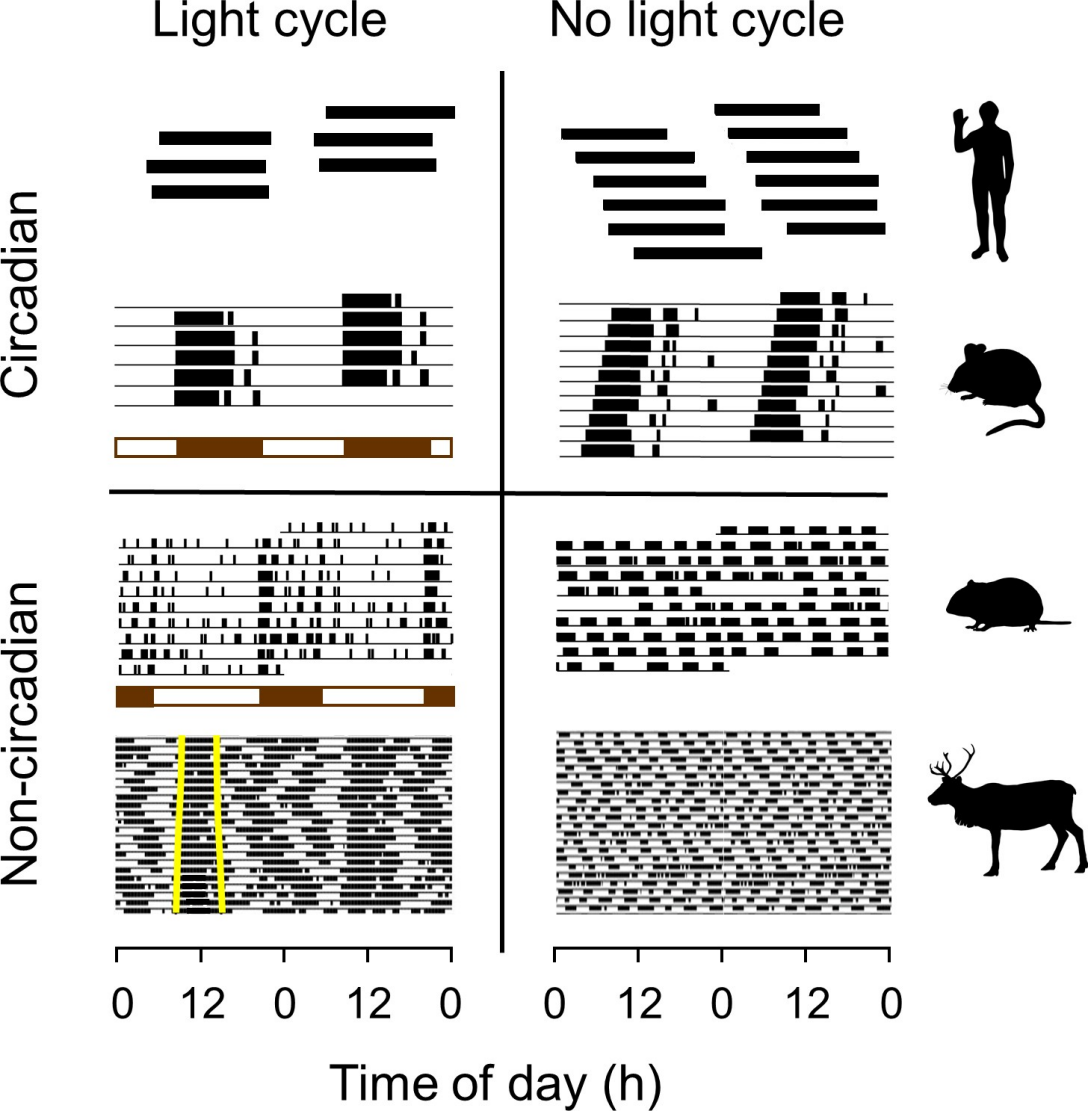
Circadian organisation is not ubiquitous in mammals. Activity patterning in (from the top) humans, mice (*Mus musculus*), voles (*M*. *arvalis*), and reindeer (*R*. *tarandus*) under 24-h LCs and NLCs. All four species display pronounced 24-h rhythms of activity under LC. These rhythms persist under NLC in humans and mice but not in voles and reindeer. Data for humans are from bunker experiments in which subjects were initially exposed to changes in light intensity synchronised to the solar day (LC) and then allowed to free-run with only self-imposed changes in light level (NLC [6]). For mice and voles, experimental light and dark phases are represented by horizontal white and brown bars, respectively. For the reindeer, free-living in their natural environment, natural photoperiod (onset and offset of civil twilight) is indicated by vertical yellow lines on the first day of each actogram, and the NLC regime was the polar night at 78° north latitude. Data for one individual of each species under each light regime are presented as double-plotted actograms. Black bars represent activity. LC, light–dark cycle; NLC, no-light cycle. *Redrawn from [6–9]*.

Maintenance of thermal balance is a major determinant of temporal activity patterns in small mammals [10], for which, moreover, the world is generally a cold place. Thus, although hyperthermia may be a problem in some environments (e.g., hot deserts), the mean surface temperature of the earth (14°C [11]) is substantially lower than the lower critical temperature (*T*_lc_) of small mammals (<100 g; median *T*_lc_ = 29°C, range = 20 to 36, *n* = 218 species [12,13], Fig 2). Such creatures are obliged to sustain a high metabolic rate simply to maintain body temperature (*T*_b_; median *T*_b_ = 37°C, range = 31 to 40, *n* = 312 species [14]), and the temporal pattern of their activity is consequently dominated by the conflicting objectives of minimising heat loss and obtaining fuel (food) to service their metabolic requirement [15].

**Fig 2:**
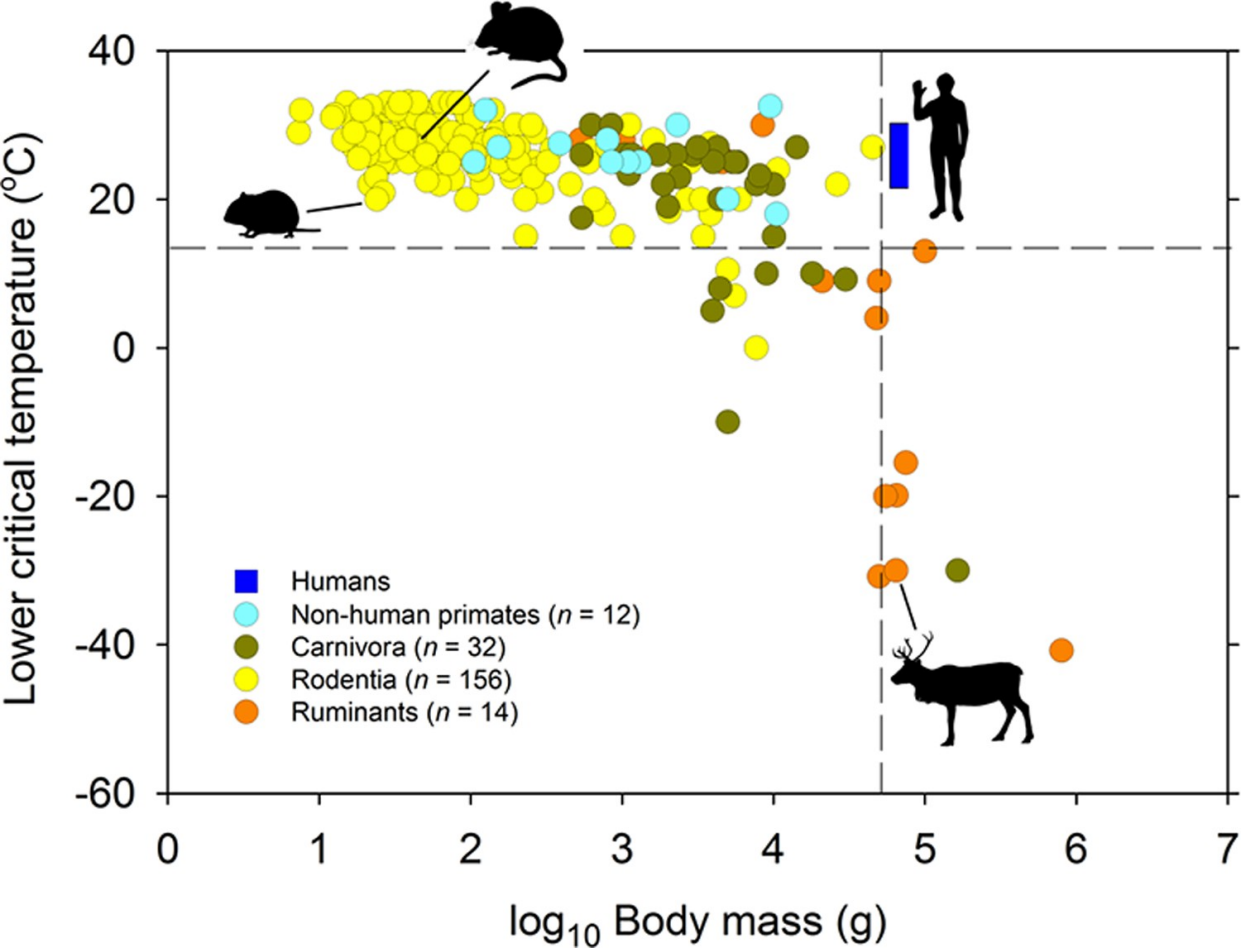
Relationship between *T*_lc_ (°C) and body mass (g) in mammals. The *T*_lc_ is defined as the ambient temperature below which the rate of metabolic heat production must be increased in order to maintain homeothermy. Of all species with *T*_lc_ above the global mean surface temperature (14°C, horizontal dashed line; [11]), humans (*T*_lc_ range from 23 to 33°C; [16,17]) are the most massive, and of all species with a body mass above 50 kg (vertical dashed line), humans have the highest *T*_lc_. For clarity, the figure includes only data for a limited number of taxa (humans, Carnivora, nonhuman primates, Rodentia, ruminants). However, the shape of the relationship between body mass and *T*_lc_ does not change when data for other groups are included (Chiroptera, Cingulata, Dasyuromorphia, Diprotodontia, Erinaceomorpha, Eulipotyphla, Hyracoidea, Lagomorpha, Macroscelidea, Monotremata, Peramelemorphia, and Soricomorpha). Species data indicated by silhouettes are, clockwise from the left, for voles (*M*. *arvalis*), mice (*M*. *musculus*), humans, and reindeer (*R*. *tarandus*). Data from [12,13,18,19]. *T*_lc_, lower critical temperature.

Small mammals commonly respond to thermal challenge (hot or cold) by withdrawing into a nest, burrow, or other place of concealment, in which they remain during the inhospitable phase of the day before reemerging and resuming activity [10]. The strategy of ‘avoidance through withdrawal’, however, presents a problem: the better a shelter insulates an animal from inhospitable environment, the less information the animal receives about the current state of that environment and, hence, about when to emerge and resume its activity. The situation is exacerbated when the animal, protected in its shelter, enters torpor or sleep. The solution has been evolution of an internal representation of the passage of time (i.e., the circadian clock), which renders the timing of reemergence independent of environmental cues. We suggest that avoidance through withdrawal has been a major factor in the evolution and maintenance of strong circadian organisation in small mammals.

The situation for large mammals (>10 kg) is quite different. By virtue of their size, these animals escape the thermal imperative to isolate themselves from the surface environment during periods of inactivity. The median *T*_lc_ of large mammals (10–800 kg) is 10 (range from −41 to +28) °C, *n* = 26; [12,13,19]), which is 4°C below global mean surface temperature (Fig 2). Moreover, most large mammals spend their entire lives above ground, where they are continuously exposed to temporal information contained in the daily cycle of light intensity: light levels increasing after a period of darkness or decreasing after a period of daylight are reliable indicators of the approach of day or night, respectively. There is therefore no reason to infer selective pressure on such animals to maintain internal representation of the passage of solar time in order to schedule the 24-h pattern of their activity. Consistent with this, there is increasing evidence that large ungulates possess weak circadian mechanisms (*Rangifer* [20–23], red deer [*Cervus elaphus*] [24], horse [*Equus ferus caballus*] [25]).

Absence of strong circadian organisation does not, however, imply absence of strong temporal organisation. Most ungulates display highly organised patterns of behaviour consisting of alternate ultradian bouts of activity (chiefly foraging) and inactivity (chiefly digestion and sleep) that persist in continuous sequence across the 24-h cycle. This is evident in boreal species under continuous photic conditions (Fig 1; see also [26]) and also in equatorial species exposed to a strong daily cycle of light intensity (wildebeest [*Connochaetes taurinus*] [27]; elephant [*Loxodonta africana*] [28]; buffalo [*Syncerus caffer*] [29]). This behaviour has been modelled in cattle as a rumen oscillator from which derives a suite of predictable relationships between the dimensions of the gut, forage digestibility, and period of the feeding rhythm [30,31].

Predominantly ultradian organisation of activity is not, however, unique to large ungulates. Voles, too, depend on microbial fermentation to extract energy from the cell walls of the plants they eat, and the resulting low rate of energy uptake likewise obliges them to engage in frequent bouts of feeding to meet their metabolic requirements [32]. These small (<30 g) animals display clear ultradian organisation in the wild, with short bouts of activity spaced at intervals of 2–4 h across the 24-h cycle (*M*. *arvalis* [33], *Microtus agrestis* [34]), and they continue to display free-running ultradian rhythms when maintained in continuous darkness (Fig 1).

Nor is ultradian organisation unique to herbivores. Shrews (Insectivora) eat invertebrates [35] and display ultradian organisation both in the wild (*Neomys fodiens*, live body mass [LBM] 17 g, [36]) and in the laboratory (*Blarina brevicauda*, LBM 20 g [37]; see also [38,39]). The small size of these creatures means that they have both a high mass-specific metabolic rate and a very small stomach [40]. The energy gained from each small meal is therefore quickly consumed, and consequently, shrews (like voles) have to replenish with frequent short (0.1–3.0 h) bouts of feeding distributed more or less evenly across the 24-h cycle [41].

These examples illustrate ways in which metabolism and alimentary function constrain temporal organisation of activity. This is not to say that the temporal pattern of activity of such species is independent of the daily cycle of light intensity. Rather, the influence of light on activity depends on the current ecological and physiological settings. Thus, the prominent peaks of crepuscular activity in ruminants are sustained by the transitions in light level at dawn and dusk and vanish when the amplitude of the light-intensity cycle is reduced naturally around the solstices (*Rangifer* [9,21]) or experimentally in the laboratory (sheep [*Ovis aries*] [42]). The coupling of activity to the light-intensity cycle evident during equinoctial periods has been attributed in *Rangifer* to direct effects of light that act through a photoperiod-dependent trade-off between predation hazard and energy balance [43]. The ultradian pattern of activity in voles, likewise, is coupled to the daily cycle of light intensity. Increasing light levels at dawn directly suppress nocturnal activity and synchronise the first daytime bout between individuals [41,44]. This is considered part of an antipredator strategy [33], and for this purpose, voles (like *Rangifer*) seem to exploit conscious assessment of risk, directly reliant on visual cues, rather than circadian entrainment [43,45]. Hence, it seems that a range of interacting factors (thermo-energetics, alimentary function, hazard) militate for or against expression of circadian rhythmicity, and the relative influence of each varies within and between species.

The question therefore arises whether strong circadian organisation should be interpreted as an ancestral feature that has become vestigial in some groups or as a derived specialisation. Modern eutherian mammals are believed to have descended from nocturnal ancestors [46] in which exploitation of darkness, possibly in response to predation by diurnal sauropsids, was facilitated by the evolution of photoreceptive systems adapted to low light and of endothermy [47–50]. Ancestral mammals were generally rather small [51,52] and so would presumably have experienced the same thermo-energetic constraints as modern small mammals. Indeed, daily torpor is an energy conservation strategy seen predominantly in ancient mammalian lineages [53]. We suggest that the adoption of a nocturnal lifestyle and avoidance through withdrawal together favoured the evolution of circadian dominance. This ancestral characteristic has been lost for those mammals in which changes in physiological and ecological constraints have removed the need for avoidance through withdrawal (large ungulates) or, alternatively, have intensified the need to feed at an ultradian frequency (voles and shrews).

Secondary loss of circadian dominance has not to date been linked to mutations at the level of the canonical ‘clock genes’, which serve as the master controllers of circadian organisation [1]. Detailed characterisation of clock genes in sheep has revealed no distinctive features in terms of DNA sequence, RNA expression cycles under a daily light-intensity cycle, protein–protein interactions, DNA binding, or transcriptional control [54]. Similarly, a survey of the genome of *Rangifer* [55] reveals both a full complement of clock genes and a high degree of sequence conservation between the coding and upstream promoter region in these and their homologues in rats, mice, sheep, and humans (personal communication, A. West to D. Hazlerigg). In voles, ultradian patterning of behaviour is associated with arrhythmic expression of clock genes in the liver, whereas gene expression rhythms in the suprachiasmatic nucleus (SCN) follow the light–dark cycle [56], and in blind mole rats (*Spalax ehrenbergi*), poorly organised activity patterns under constant light conditions are associated with low-amplitude rhythms of circadian gene expression in the brain [57]. The best-documented function of clock genes in ungulates relates to seasonal timekeeping for which measurement of day length (photoperiod) is the key attribute. Here, however, light controls the expression of clock genes directly, via the hormone melatonin and without circadian gating, and hence drives seasonal changes in physiological and behavioural function [58–60]. Furthermore, the presence of so-called clock genes in the absence of robust circadian organisation may reflect the importance of their molecular functions for biological processes that have nothing to do with timekeeping per se (e.g., casein kinase 1 role in wnt signalling [61], cryptochrome role in magnetic sensitivity [62], bmal1 role in hypoxia sensing [63]).

It is also important to distinguish between the presence and the efficacy of a trait. Demonstration of a circadian pattern of activity in an organism maintained under laboratory conditions provides no information about its temporal organisation of activity under natural conditions. Voles provide a striking example: voles living in cages furnished with running wheels normally display a strongly nocturnal pattern of activity. This pattern, moreover, may free-run in constant darkness, thus bearing the hallmark of circadian organisation [7,41,64], yet it derives specifically from the presence of the running wheel: voles living in cages without running wheels maintain their natural ultradian pattern [56]. The reasons for this effect are unknown, but running in wheels may be intrinsically rewarding [65,66]: indeed, rats, mice, shrews, and other wild creatures voluntarily climb into and run in wheels placed outside in the field [67]. Furthermore, wheel running distances—up to several kilometres in a single episode—are sensitive to energetic status [68], and making voles or mice ‘work for food’ by coupling wheel running to their food supply induces ultradian activity ([69,70] and personal communication, R.A. Hut to D. Hazlerigg). The emergent picture is one in which temporal organisation depends on complex energetic and other ecological constraints, and the running wheel is a device that artificially emphasises the circadian component.

Running wheels also corrupt our view of behavioural organisation because running rodents appeal to anthropomorphic perception of biological timekeeping. Just as voles may be considered miniature cows [71], so humans are often considered massive mice. This is essentially the precept upon which biomedical science is based, and its general worth has been proved innumerable times. Mouse models afford us insights into many aspects of human health and disease, and the analogy extends to the circadian system: despite being three orders of magnitude heavier, humans, like mice, withdraw for consolidated periods of sleep and show robust circadian rhythmicity [72]. The resolution of the paradox of strong circadian organisation in humans may lie in their unusually low thermal tolerance compared with similar-sized mammals (Fig 2). The *T*_lc_ of humans is similar to that of mice (*T*_lc_ of humans 23–33°C [range], *T*_lc_ of mouse 29°C [16,17]) and is more than 40°C higher than the median for mammals with a body mass of 50 kg or more (Fig 2). All primates have low thermal tolerance, which suggests that this is an ancestral feature (Fig 2); this and the associated need for avoidance through withdrawal during rest may at least in part account for human circadian organisation.

The temporal organisation of mammals cannot be dissociated from their ecological environment or from the trade-offs that constrain their behaviour. Metabolic and alimentary constraints can render the thermal benefit of circadian withdrawal inaccessible to a small mammal. A large mammal with unrestricted access to temporal cues may organise its behaviour directly without reference to a circadian clock. In cases like these, circadian timekeeping seems to have become vestigial. In arriving at this view, we emphasise the importance of behavioural organisation, and we see little reason to anticipate internal physiological or molecular circadian rhythmicity in organisms in which behaviour is not circadian. Unfortunately, studies addressing this issue are rare, reflecting the stultifying effect of ascribing a ‘circadian’ basis to approximately 24-h rhythms observed only under light–dark cycles. There is clearly a need to look deeper, and it is timely to do so given the ease with which gene expression can now be both monitored and manipulated in nonmodel species. Only by so doing in an unbiased way across taxa can we reach a properly nuanced view of the importance of circadian organisation in mammals.

## Abbreviations

LBM: live body mass
LC: light-dark cycle
NLC: no-light cycle
SCN: suprachiasmatic nucleus
*T*_b_: body temperature
*T*_lc_: lower critical temperature
